# Predicting the impacts of climate change on the distribution of threatened forest-restricted birds in Madagascar

**DOI:** 10.1002/ece3.497

**Published:** 2013-02-15

**Authors:** Rado H Andriamasimanana, Alison Cameron

**Affiliations:** 1 Asity MadagascarP.O. Box 1074, Antananarivo, Madagascar; 2 School of Biological Sciences, Medical Biology Centre, Queen's University Belfast97 Lisburn Road, BT9 7BL, Belfast, United Kingdom

**Keywords:** Climate change, extent of occurrence, forest-restricted birds, habitat suitability, Madagascar, maximum Entropy, niche modeling, threatened species

## Abstract

The greatest common threat to birds in Madagascar has historically been from anthropogenic deforestation. During recent decades, global climate change is now also regarded as a significant threat to biodiversity. This study uses Maximum Entropy species distribution modeling to explore how potential climate change could affect the distribution of 17 threatened forest endemic bird species, using a range of climate variables from the Hadley Center's HadCM3 climate change model, for IPCC scenario B2a, for 2050. We explore the importance of forest cover as a modeling variable and we test the use of pseudo-presences drawn from extent of occurrence distributions. Inclusion of the forest cover variable improves the models and models derived from real-presence data with forest layer are better predictors than those from pseudo-presence data. Using real-presence data, we analyzed the impacts of climate change on the distribution of nine species. We could not predict the impact of climate change on eight species because of low numbers of occurrences. All nine species were predicted to experience reductions in their total range areas, and their maximum modeled probabilities of occurrence. In general, species range and altitudinal contractions follow the reductive trend of the Maximum presence probability. Only two species (*Tyto soumagnei* and *Newtonia fanovanae*) are expected to expand their altitude range. These results indicate that future availability of suitable habitat at different elevations is likely to be critical for species persistence through climate change. Five species (*Eutriorchis astur, Neodrepanis hypoxantha, Mesitornis unicolor, Euryceros prevostii,* and *Oriola bernieri*) are probably the most vulnerable to climate change. Four of them (*E. astur, M. unicolor, E. prevostii,* and *O. bernieri*) were found vulnerable to the forest fragmentation during previous research. Combination of these two threats in the future could negatively affect these species in a drastic way. Climate change is expected to act differently on each species and it is important to incorporate complex ecological variables into species distribution models.

## Introduction

Madagascar's avifauna is characterized by high rates of endemism and dependence on forest habitats. Five families are endemic to the region. Madagascar shares Leptosomatidae and Vangidae families with the Comoro islands, but three families, Mesitornithidae, Brachypteraciidae, and Bernieridae are limited only to Madagascar (Hawkins and Goodman 2003). Thirty three (33) of 37 endemic genera are limited to forested habitats. Of the 204 species breeding on Madagascar, 120 (59%) are endemic to the region. Rates of endemicity are highest among the forest dependent species, at 91% (Morris and Hawkins 1998), and due to historical anthropogenic deforestation, almost 50% of the threatened species with IUCN status are forest-restricted species.

As elsewhere, until recently, conservation concern in Madagascar has mainly focused on habitat loss, but during the last decade, concern over global climate change has increased. Examples from other parts of the world (Phillips et al. 2006; Phillips and Dudíck 2008; Raphael 2008) show that we can expect the effects to differ across species depending on their respective biology and ecology. Therefore, the objective of this research was to assess the potential impacts of climate change on the distribution of threatened forest-restricted bird species in Madagascar, in order to inform strategies for bird conservation in Madagascar in the face of global climate change.

In this study, we first investigate the effects of data limitations for modeling, exploring the potential use of pseudo-absence data and the relative importance of climate and non-climate variables, before assessing the impact of the climate change on our focal group of species.

## Materials and methods

Geo-referenced occurrence data (observation data accompanied by accurate records of latitude and longitude) for 17 threatened (BirdLife International 2008), forest-restricted, endemic, bird species were obtained from Asity Madagascar (Table 1). We obtained field observation data from the Important Bird Area assessment project (Projet ZICOMA, 1999), which was active from 1996 to 1999. As no absence data were available, Maximum Entropy (MaxEnt version 3.3.2) (Phillips et al. 2004, 2006; Phillips and Dudíck 2008; Elith et al. 2011) was used to model individual species distributions. Maxent is among the highest-performing group of programs utilizing presence-only data (Elith et al. 2006; Hijmans and Graham 2006; Phillips and Dudíck 2008).

**Table 1:** List (in taxonomic order) of the threatened forest-restricted birds used for the research

Species	Records	UICN Status
Eutriorchis astur*	10	Endangered
*Mesitornis variegata*	5	Vulnerable
*Mesitornis variegata*+	1	Vulnerable
Mesitornis unicolor*	14	Vulnerable
*Monias benschi*	1	Endangered
Tyto soumagnei*	7	Endangered
Brachypteracias leptosomus*	25	Vulnerable
Brachypteracias squamigera*	18	Vulnerable
*Uratelornis chimaera*	2	Vulnerable
Neodrepanis hypoxantha*	13	Vulnerable
*Calicalicus rufocarpalis*	5	Vulnerable
Oriolia bernieri*	11	Vulnerable
*Xenopirostris damii*	2	Endangered
Euryceros prevostii*	10	Vulnerable
Newtonia fanovanae*	7	Vulnerable
*Monticola erythronotus*	1	Endangered
*Bernieria apperti*	3	Vulnerable
*Bernieria tenebrosus*	3	Vulnerable

* Species used to test the importance of the forest cover variable.

+ *Mesitornis variegata* is the only record of the species in the eastern forest of Madagascar (Special Reserve Ambatovaky).

Current and future climate variables were sourced from http://www.worldclim.org, at 30 arc-second resolution. For the future scenario, we selected the HadCM3 (Hadley Centre, United Kingdom) model for the IPCC3 B2a scenario (Randall et al. 2007) that was widely available during the time of the research. This scenario predicts a 2°C increase in global mean temperature by 2050. We used six variables directly (mean temperature of the coldest month, mean temperature of the hottest month, mean annual temperature, mean precipitation of the wettest month, mean precipitation of the driest month, mean annual precipitation) and derived three additional variables (annual evapotranspiration, annual water balance, and the number of months with a positive water balance). We utilized a map of forest cover change from the period 1950–2000 produced by Conservation International (Harper et al. 2007) at 28.5-m resolution to develop forest cover layers for 1990 and 2000. We summarized the finer resolution (28.5 m) forest cover layer to provide percent forest cover within the same 30 arc-second grid as the climate variables. We used combinations of current climate and forest cover, and future climate and current forest cover variables to predict species distributions for the years 2000 and 2050. These climate and forest cover variables are described in detail by Kremen et al. (2008).

First, to examine the importance of including forest cover as a modeling variable, we compared the predictive power of two sets of Maxent models; one set produced using only the climate variables, and a second set using the climate variables plus the forest cover variable. As our locality data are from Important Bird Area inventories conducted between 1997 and 1999, we used forest cover for 2000 for this test. We tested the effects of including forest cover as a modeling variable for nine species, which have six or more occurrence records (Table 1). We chose species with six or more records because Maxent has proven to perform well on small samples (Hernandez et al. 2006), and Jackknife tests on Maxent models of geckos in Madagascar showed significantly high success rates where six or more localities were available (Pearson et al. 2007).

We partitioned our data for training and testing prior to use in Maxent in order to ensure that the same training and testing partitions were used in the two sets of models. We run MaxEnt using the default settings, except that we used user-defined training and testing data, and constrained MaxEnt from predicting into climate conditions outside the limits encountered during training by disabling the extrapolation option (Phillips et al. 2006).

We generated model test statistics by partitioning 75% of the data for model training and using the remaining 25% of the data for model testing (Phillips et al. 2006). This was repeated four times for fourfold cross-validation. Each species model was run with and without the forest layer, using exactly the same four training and testing data sets for both runs per species. We compared for each species the means of the four Area Under the receiver operator Curve (AUC) values (Fielding and Bell 1997), for the models that included and excluded forest cover.

As eight species (Table 1) had insufficient occurrence records to model their distributions, we tested the potential to model the effects of climate change using pseudo-presences by comparing models built from real-occurrence records with models built from pseudo-presences for the nine more common species, with six or more occurrences.

We obtained extent of Occurrence (EOO) polygons for all 17 species from the taxonomic working group responsible for the prioritization of the conservation areas in Madagascar. This study was completed within the framework of the extension of the protected area network in Madagascar (Razafimpahanana et al. 2008). During workshops, regional ornithological experts validated, and where necessary updated, EOO polygons and then refined them using species-specific habitat information (Harper et al. 2007) and altitudinal range limits. Pseudo-presences data were extracted once from within these Refined Extent of Occurrence (REOO) polygons using random function in a Geographical Information System program. We split these data into four set of 25% and used them as presences in MaxEnt.

To provide rigorous performance comparisons, we implemented another fourfold cross-validation procedure, similar to those above. This time the four different pseudo-presence data sets were used to train the models and the four data files each containing 25% of the real-presence data were used for testing. The process was repeated four times for the fourfold cross-validation. All nine climate layers and the forest cover layer were used. The four AUC's per species were averaged, and the average for each species was compared with the average from the four models already produced with real-occurrence data above. If the mean AUC values from the pseudo-presence models were lower than those from the real-presence models, this indicates that the pseudo-presence data produce worse models and that pseudo-presence data are not a good surrogate for modeling the distributions of species with too few real-occurrence records.

Finally, in order to assess the potential impact of climate change on the nine species that had six or more occurrence records, we derived models using all of their real-presence data and were projected to 2000 and 2050 climate conditions. For the each species' 2000 and 2050 models, the following three comparisons were made: the number of thresholded grid cells, the maximum logistic model value, and the minimum and maximum altitudes within the thresholded model area. To be able to do this comparison, the continuous data from the models have to be converted into binary data. We transformed then the 2000 and 2050 models from Maxent's logistic output (continuous values between 1 and 0) to presence–absence models (binary values, of 1 and 0) using species specific model threshold values. To set each species model threshold level, the minimum model value was selected from across the species real-occurrence locations.

The thresholded Maxent models were intersected with altitude from a 1-km digital elevation model (USGS 2004) and we calculated predicted changes in minimum and maximum altitude limits between 2000 and 2050 for each species.

We performed Shapiro-Wilk test (Shapiro and Wilk 1965) to test the normality of the data sets (thresholded area change, altitude range change, and change in maximum probability of occurrence). Following this, if the data were normal, we conducted Student's *t*-tests (Fowler and Cohen 1990) to compare the 2000 and 2050 results. If the data were not normal, we did Mann–Whitney *U*-tests.

Finally, we conducted correlations between thresholded area change, altitude range changes, and changes in maximum probability of occurrence across species.

We used the following criteria to classify the species. If the thresholded model area is predicted to decline more than 75% of those in 2000 and at the same time, the maximum presence probability predicted model value is also predicted to decline more than 50%, the species is evaluated as at highest risk. If the area expands and the maximum presence probability raise more than 25%, the species is of lowest concern.

## Results

The AUC results for the models using climate variables and the additional forest cover variable are significantly higher than those from the models using climate variables only (*t*-test, *t* = 5.92, df = 8, *P* < 0.05) (Table 2). Due to this significant performance difference, all further comparisons were based on models using the climate and forest cover variables.

**Table 2:** Averages of AUC values from fourfold cross-validation of models using only climate variables, and models using climate variables and forest cover (2000). All models are derived from real-presence data

Species	Without forest		With forest	
	
	Mean AUC	Standard deviation	Mean AUC	Standard deviation
*Brachypteracias leptosomus*	0.900	0.031	0.948	0.017
*Brachypteracias squamigera*	0.909	0.031	0.940	0.023
*Euryceros prevostii*	0.894	0.035	0.919	0.036
*Eutriorchis astur*	0.876	0.032	0.927	0.033
*Mesitornis unicolor*	0.921	0.026	0.932	0.033
*Neodrepanis hypoxantha*	0.922	0.033	0.953	0.020
*Newtonia fanovanae*	0.866	−0.199	0.918	−0.210
*Oriolia bernieri*	0.911	0.016	0.929	0.033
*Tyto soumagnei*	0.819	−0.220	0.889	−0.206

The AUC's for the next two sets of models for the nine species, derived from real-presence data or from pseudo-presences, also differ significantly (*t*-test, *t* = 7.847, df = 8, *P* < 0.05), with the models built from the real-occurrence data being higher (Table 3).

**Table 3:** Comparison of the AUC values of nine species modeled using real presences and pseudo-presences randomly extracted from refined extent of occurrence polygons

Species	Real presence (with forest)		Pseudo-presence (with forest)	
	Mean AUC	Standard deviation	Mean AUC	Standard deviation
*Brachypteracias leptosomus*	0.900	0.031	0.602	0.114
*Brachypteracias squamigera*	0.909	0.031	0.463	0.127
*Euryceros prevostii*	0.894	0.035	0.606	−0.408
*Eutriorchis astur*	0.876	0.032	0.386	−0.497
*Mesitornis unicolor*	0.921	0.026	0.363	0.150
*Neodrepanis hypoxantha*	0.922	0.033	0.826	0.082
*Newtonia fanovanae*	0.866	−0.199	0.437	−1.000
*Oriolia bernieri*	0.911	0.016	0.417	0.052
*Tyto soumagnei*	0.819	−0.220	0.225	−1.000

**Figure 1:**
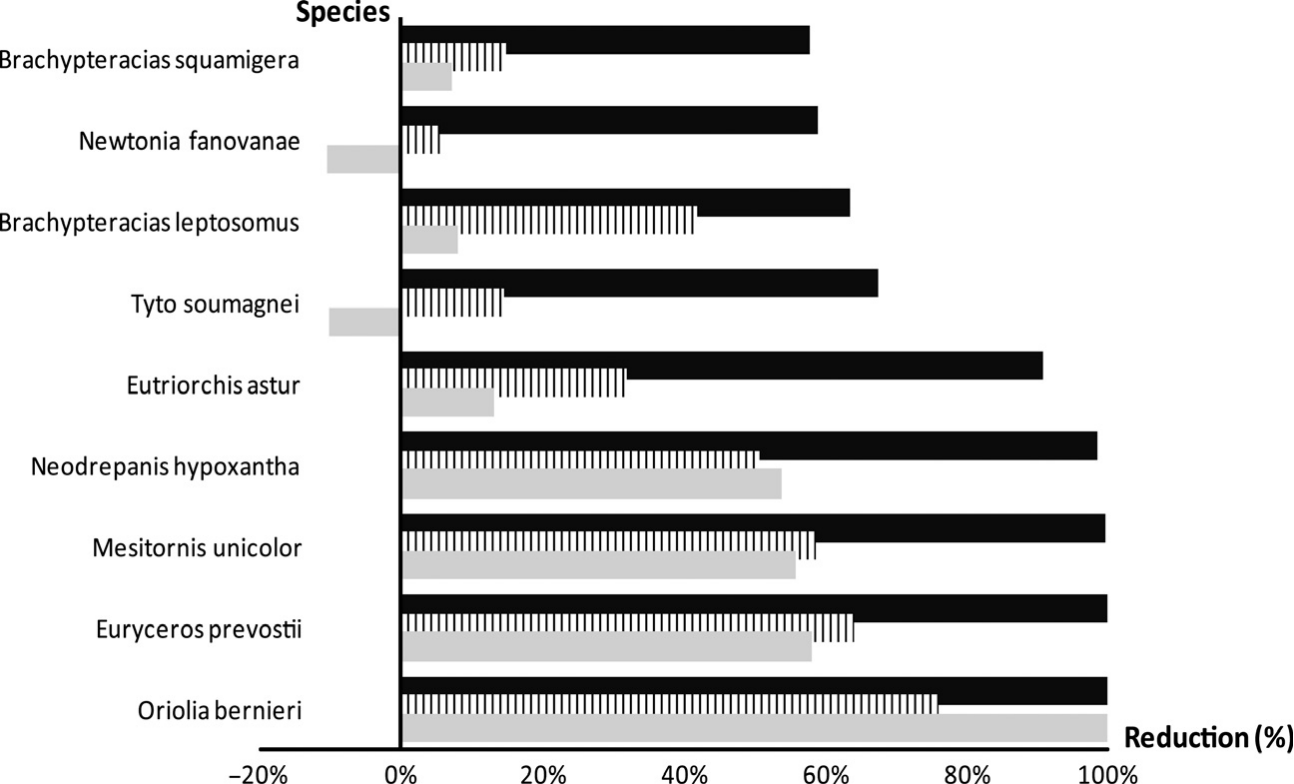
Percentage of the area (number of 30 arc-second grid cells) [in plain black], presence probability [in stripped white], and altitude [in gray] reduction in the thresholded species models for 2000 and 2050. Models derived from real-presences data of the nine species.

Reduction in the ecological niche of all the nine species was predicted to vary from 58% to 100% (Fig. 1). Differences of the areas of the models in 2000 and the predicted models were significantly inferior to those of the models in 2000 (*U*-test, *U* = 8, df = 8, *P* < 0.05). Five species (*Euryceros prevostii, Mesitornis unicolor, Oriolia bernieri, Neodrepanis hypoxantha,* and *E. astur)* have been predicted to lose more than 90 percent of their ecological niche (Fig. 1). There was no case of expansion in new places, the predicted reductions are all inside the original (2000) ecological niche. So, the predicted left area overlaps with the original ecological niche.

All species are predicted to have their maximum presence probability decreased. Decrease of the maximum probability presence ranged from 6% to 76% (Fig. 1). The differences between the values of the maximum presence probability of the models in 2000 and those of 2050 are significant (*t*-test, *t* = 4.95, df = 8, *P* < 0.05). There is also a strong positive correlation between the area reduction and that of the maximum presence probability (Pearson correlation = 0.85). The same result is seen between the altitudinal range and the maximum presence probability (Pearson correlation = 0.93).

Six species have their altitudinal range diminished in 2050. Two species (*Newtonia fanovanae* and *Tyto soumagnei*) have only their maximum expanded (Fig. 1). The difference of the altitudinal range variations between the models in 2000 and those of 2050 is significant (*U*-test, *U* = 7, df = 7, *P* < 0.05). Three (3) species (*N. hypoxantha, M. unicolor,* and *E. prevostii*) have their range of altitudes restraint considerably from both limits (Table 4). Three more species (*E. astur, Neodrepanis hypoxantha,* and *Tyto soumagnei*) are predicted to move their minimum altitude higher and only one species (*Brachypteracias squamigera*) is predicted lower from its maximum altitude (Table 4).

**Table 4:** Area (number of 30 arc-second grid cells) presence probability and altitude variations of the thresholded species models for 2000 and 2050. Models derived from real-presences data of the nine species

Species	Thresholded model area (pixel count)		Maximum probability of occurrence		Altitude range (m)	
		
	2000	2050	2000	2050	2000	2050
*Oriolia bernieri*	99 841	0	0.894	0.203	0–2744	1456–2405
*Euryceros prevostii*	136 251	41	0.904	0.337	0–2744	999–2568
*Mesitornis unicolor*	148 966	436	0.929	0.337	0–2744	1551–2506
*Neodrepanis hypoxantha*	73 147	1270	0.951	0.475	12–2745	1145–2744
*Eutriorchis astur*	112 149	6867	0.909	0.592	0–2744	609–2744
*Tyto soumagnei*	112 377	31 813	0.92	0.761	0–2744	242–2744
*Brachypteracias leptosomus*	102 898	38 622	0.993	0.498	0–2744	3–2744
*Newtonia fanovanae*	148 260	70 430	0.887	0.848	0–2744	1–2744
*Brachypteracias squamigera*	79 047	43 734	0.993	0.83	0–2744	0–2405

Height species (*Monias benschi, Uratelornis chimaera, Calicalicus rufocarpalis, Mesitornis variegata, Xenopirostris damii, Bernieria tenebrosus, Monticola erythronotus, and Bernieria apperti*) could not be modeled due to the insufficiency of the number of the occurrences.

## Discussion

Modeling based on the presence data allowed us to assess the effect of the climate variability for nine threatened forest endemic bird models in a standardized way. As we assume that forest cover will not change in the future, the modeled predictions are based only on potential climate change. If deforestation continues at its historical pace in Madagascar, then this will result in even larger distribution changes for these 17 species, elevating their threatened status even further.

Without the forest cover variable, we have observed that distributions are predicted in areas outside the species known habitats and ranges. Knowing that species are forest dependent, we attempted to reduce the predicted area outside of forest by including the forest. The significance of AUC results for models with the forest variable confirms the affinity of the species to the forest habitat. We decided to include forest cover for the rest of the analysis based on this evident consistent improvement in AUC values.

Our tests demonstrated that modeling using pseudo-presence models was not accurate. The probable reason is that all habitats inside the extent of occurrence are not suitable for the species (IUCN 1994).

Following criteria we have set, *E. astur, N. hypoxantha, M. unicolor, E. prevostii,* and *O. bernieri* are species that we consider most threatened by climate change following real-presence data analysis (Table 5) They are predicted to have lost over 90% of original ecological niche in 2050. The chance of these species to find their preferred environmental conditions is very low, except for *E. astur*. Furthermore, the chance of this species to survive in the 5% of their ecological niche remaining is very low.

**Table 5:** Threat level of each species following the criteria fixed on the thresholded model area and the maximum presence probability

Species	Threat level
*Oriolia bernieri*	Most vulnerable
*Euryceros prevostii*	Most vulnerable
*Mesitornis unicolor*	Most vulnerable
*Neodrepanis hypoxantha*	Most vulnerable
*Eutriorchis astur*	Most vulnerable
*Tyto soumagnei*	Less vulnerable
*Brachypteracias leptosomus*	Less vulnerable
*Newtonia fanovanae*	More adapted
*Brachypteracias squamigera*	More adapted

*Brachypteracias leptosomus* and *Tyto soumagnei* are the species mostly at risk after the five preceding species. These species would lose about 62% and 72%, respectively, of their original ecological niches. Inside the two-thirds of the ecological niche predicted, the probability of *Brachypteracias leptosomus* to find his suitable environmental conditions would be reduced by 50%. The chance of *Tyto soumagnei* to find its ecological conditions in a quarter of its ecological niche predicted that 2050 would be affected by slight decrease (17%).

Species which are likely able to adapt to climate change would be *Brachypteracias squamigera* and *Newtonia fanovanae*. They would keep in 2050 around half of their ecological niches in 2000. The presence probability within their modeled distribution in 2050 would be affected slightly.

As prediction models in 2050 are all within the original models, elevation changes in 2050 should not be a problem assuming that the connectivity between forests along the altitudinal gradients will be kept. We found strong correlations among the distribution restriction, the difficulty to find adequate ecological condition, and the altitudinal restraint. Species which are predicted vulnerable to climate change have their altitudinal margin restricted and have a difficulty to find their favorable ecological condition in the future. This does not concern *Newtonia fanovanae* and *Brachypteracias leptosomus* as no changes were noted from their original range of altitudes.

Andriamasimanana et al. (2001) have carried out a study of the effect of the fragmentation on species occurring to the eastern forest of Madagascar. The results of this research were very similar to the current research. *E. astur, M. unicolor, E. prevostii,* and *O. bernieri* were found absent once there is forest fragmentation. *Brachypteracias leptosomus* and *B. squamigera* were sensitive, but more frequent in fragmented forests. *Newtonia fanovanae* was contrary very vulnerable to the forest fragmentation. The six-first species would be very threatened if there is a combined effect of fragmentation and climate change in the future.

Briefly, this is the first attempt to assess the impacts of climate change on this taxon in Madagascar. We demonstrate that using forest cover in addition to climate variables produces better predictions of the species distributions. Climate change on its own is demonstrated to have considerable impacts on the five threatened species, and it is essential that further deforestation is prevented to minimize the threat from climate change to these species. Contributions of the climate variables and the forest variable vary considerably from one species to another. For better analysis of the climate impact, contribution of the climate variables on each species and its change in time should be examined. The climate variables which contributed more to the modeling should be identified and the species modeling should be tuned up using only these variables. Climate variables used to model the ecological niche should vary from one species to another. This was confirmed with the case of *M. unicolor*. If we compare the latter species to its similar species (e.g., *Brachypteracias squamigera*) in the eastern forest, its similar species would suffer more in 2050. However, this was not the case. It is then very important to analyze what climate variable the species depends on and how this variable will change in the course of time. For restricted-range species like *Monticola erythronotus* and *Bernieria apperti* also*,* the analysis should be done at higher scale (e.g., site scale) to have more precise environmental data. Monitoring and analyses to determine the effects of the contribution of each individual climate variable on the species model are recommended (Araújo and Guisan 2006). Ecosystem approach using, in addition to the current variables, other environmental variables (e.g., geology, soil type) should be experimented. Species ecology (dependence to a specific microhabitat and diet) should be considered in the future.
